# Novel halotolerant microbial system enhanced by gamma irradiation for advanced multimetal bioremediation

**DOI:** 10.1186/s12866-026-05728-8

**Published:** 2026-11-05

**Authors:** Sahar M. Ouda, Samah A. Yousef, El Sayed R El-Sayed, Abdullah M. Othman, Gamal Abdelaziz

**Affiliations:** 1 Plant Research Department, Nuclear Research Center, Egyptian Atomic Energy Authority, 13759, Cairo, Egypt; 2 Egypt Second Research Reactor (ETRR-2), Egyptian Atomic Energy Authority, Cairo, 13759 Egypt; 3 Labeled Compounds Department, Hot Laboratories and Waste Management Center, Egyptian Atomic Energy Authority (EAEA), 13759, Cairo, Egypt

**Keywords:** Halotolerance, Bioremediation, Heavy metals, *Serratia proteamaculans*, *Rhodotorulamucilaginosa*, Gamma irradiation, Carotenoids, Saline ecosystems

## Abstract

**Background:**

Saline industrial effluents are difficult to treat because elevated ionic strength can inhibit biological treatment and alter metal speciation. This study evaluated two moderately halotolerant microorganisms recovered from seawater and shark-associated samples and examined whether gamma selection altered the performance of the more radioresistant isolate.

**Results:**

The isolates were identified as *Serratia proteamaculans* PX875896 and *Rhodotorula mucilaginosa* PX876763 by 16 S and 18 S rRNA gene sequencing, respectively. Both grew at 3–5% (w/v) NaCl but not at 10% or higher. In synthetic saline medium, *S. proteamaculans* showed the highest removal after 72 h for Cd (50.71%), Mo (42.56%), Cu (34.13%), and Co (25.26%); no net Cr removal was detected. *S. proteamaculans* was not recovered after 0.5 kGy, whereas *R. mucilaginosa* survived exposure up to 4 kGy. Relative to the wild yeast, gamma-selected yeast derivatives showed higher removal for several metals, including Co (22.61% at 48 h) and Cu (12.34% at 72 h) by the 4 kGy derivative and Mo (14.73% at 72 h) by the 3 kGy derivative. The 4 kGy derivative also contained more total carotenoids (67.30 µg/mL) than the wild strain (44.40 µg/mL). In textile wastewater, the 4 kGy derivative yielded the greatest removal of Mo (93.05%), Cr (77.88%),Co ( 60.80%) and Cd (51.42%), whereas *S. proteamaculans* yielded the greatest removal of Mo ( 58.43 %, Cr 57. 14 %, Fe (52.17%) and V (74.68%).

**Conclusions:**

Saline-derived Serratia proteamaculans PX875896 showed greater metal removal in synthetic saline medium, while Rhodotorula mucilaginosa PX876763 exhibited broader metal tolerance and greater gamma resistance.The 4 kGy yeast derivative improved multimetal removal from textile wastewater, coinciding with a more carotenoid-rich phenotype.These findings highlight the value of moderate halotolerance, gamma-assisted selection, and complementary bacterial–yeast approaches, while showing that tolerance alone does not predict removal efficiency. l

**Supplementary Information:**

The online version contains supplementary material available at https://doi.org/10.1186/s12866-026-05728-8.

## Introduction

 Saline and hypersaline ecosystems are chemically complex habitats in which low water activity, elevated ionic strength, and fluctuating nutrient availability select for unusually resilient microbial communities. These systems include marine salterns, coastal salt marshes, inland saline lakes, athalassohaline brines, and evaporitic pools, each shaped by its own ionic balance and hydrological history [1–4]. Although such environments were once viewed mainly as ecological curiosities, they are now recognized as valuable reservoirs of enzymes, pigments, osmoprotectants, and stress-tolerant metabolisms with direct industrial and environmental relevance [5–8].

Saline and hypersaline habitats are increasingly exposed to petroleum hydrocarbons, mixed toxic metals, dye residues, and other persistent pollutants. Because many conventional treatment systems lose efficiency as salinity rises, remediation of these matrices is a stubborn technical problem. High ionic strength can suppress microbial diversity, decrease oxygen transfer, alter pollutant speciation, and reduce the performance of standard sorbents and enzymes [9–13]. For that reason, halotolerant and halophilic microorganisms have become attractive candidates for treatment strategies tailored to saline waste streams, and recent reviews increasingly emphasize strain selection, omics-guided process design, and robust mixed systems as the key next step toward field deployment [14–18].

Heavy-metal pollution remains especially problematic because metals do not degrade and can cycle between dissolved, precipitated, sorbed, and biologically associated forms. Physicochemical treatments such as chemical precipitation, membrane separation, reverse osmosis, ion exchange, and electrochemical removal are valuable, but they often become costly or inefficient when contaminant concentrations are low, mixed, or associated with saline matrices [19–23]. Biological treatment offers a different logic: cells and their extracellular products can adsorb, accumulate, reduce, precipitate, or transform metals while operating under milder conditions [24–28].

Marine-animal-associated samples should not be treated as equivalent to seawater. Shark-associated microbial communities can differ from surrounding seawater and vary among anatomical sites and host species [29]. Likewise, cultivable yeasts recovered from marine fish include *Rhodotorulamucilaginosa*, demonstrating that animal-associated marine niches can yield stress-tolerant yeasts while remaining ecologically distinct from free-living seawater communities [30]. The present isolates are therefore described according to their measured salt-growth phenotype rather than inferred to be halophiles from their source alone.

Within this broader framework, yeasts and bacteria from saline habitats deserve particular attention. Rhodotorula spp. are notable for carotenoid formation, extracellular polymeric material, vacuolar sequestration, and broad stress tolerance, all of which can influence metal uptake and oxidative defense [31–35]. Their carotenogenic machinery is now being clarified at the genomic level, which improves the biological interpretation of stress-adapted phenotypes [36, 37]. Serratia spp., meanwhile, are metabolically versatile heterotrophs whose cell-envelope chemistry, enzyme systems, and extracellular activities can support metal binding, reduction, and survival in contaminated settings [38–40]. In saline systems, however, tolerance does not necessarily translate directly into removal efficiency, so strain-level evaluation is essential.

An additional layer of interest concerns radiation-assisted strain improvement. Ionizing radiation can severely damage cells, yet sublethal doses may also reveal rare survivors with altered physiological traits. In pigmented microorganisms, carotenoids are repeatedly linked to oxidative-stress management, membrane stabilization, and survival under ultraviolet or ionizing stress [41–45]. In Rhodotorula, stress can redirect metabolism toward carotenoid accumulation and extracellular protection systems, suggesting that irradiation-based selection may be useful for developing robust metal-removing variants [32, 46, 47]. Related precedent exists in red yeasts, where mutagenesis has been used to obtain carotenoid-hyperproducing derivatives [48]. The aim of the present work was to recover culturable microorganisms from seawater and shark-associated samples, identify selected bacterial and yeast isolates, determine their NaCl tolerance and metal susceptibility, compare their multimetal-removal performance, and test whether gamma selection altered the phenotype of the more radioresistant isolate. The final objective was practical rather than purely descriptive: to determine whether wild or gamma-selected halotolerant isolates could be moved from laboratory media to real polluted textile wastewater.

## Materials and methods

### Sampling, isolation, and purification

Seawater samples and shark tissues were obtained from a local fish market. Sterile swabs were used to sample the skin, scales, eyes, fins, and stomach surfaces. Because these were market-derived specimens, the animal-associated samples were regarded as host-associated marine material and not as proxies for seawater or hypersaline habitats. Swabs and water samples were suspended in sterile marine water and plated on nutrient agar supplemented with 3.5% total salts. Plates were incubated at 29 °C for 48 h. Colonies differing in morphology were purified by repeated streaking, and one representative colony per morphotype was retained to minimize duplicate isolation of the same strain.

### Molecular identification of selected isolate

Genomic DNA was extracted using the HiPure Bacterial and Yeast Genomic DNA Purification Spin Kit (HiMedia, India). DNA integrity was checked on 1% agarose gels and quantified spectrophotometrically. Bacterial isolates were identified by partial 16 S rRNA gene sequencing and yeast isolates by partial 18 S rRNA gene sequencing. Consensus sequences were compared with the NCBI database using BLAST. For phylogenetic placement, related sequences were aligned with ClustalW, and trees were generated in MEGA 7 using the neighbor-joining method with 500 bootstrap replicates.

### NaCl tolerance assay

Fresh cultures were streaked onto nutrient agar plates containing 3.0, 3.5, 5, 10, 15, 20, and 25% (w/v) NaCl. Plates were incubated at 29 °C for up to 5 days, and growth was recorded visually after 24, 48, 72, and 96 h.

### Heavy-metal susceptibility screening

Agar diffusion assays were used to determine the concentrations suitable for subsequent removal tests. Salt-supplemented agar plates were inoculated with each isolate, and wells were filled with graded concentrations of Co(II), Cu(II), Mo(VI), Cr(VI), and Cd(II) salts (10 to 0.078 mM). After incubation for 24 h, visible inhibition zones were measured. For the removal experiments, working concentrations were selected below the lowest concentration yielding a consistent inhibition halo.

### Metal-removal experiments in synthetic saline medium

Erlenmeyer flasks containing nutrient broth supplemented with 3.5% (w/v) NaCl and the selected metal concentrations were inoculated with 1 mL of a 12 h culture (approximately 1.5 × 10^8 cells/mL). For every metal and sampling time, an uninoculated metal-containing flask was incubated and processed in parallel as an abiotic control; isolate-only flasks without added metals were also included for pH and total dissolved solids (TDS) measurements. Cultures and controls were incubated at 29 °C on a rotary shaker at 130 rpm. Supernatants collected after 48 and 72 h were clarified by centrifugation (10000 × *g*, 20 min, 24 °C), filtered through 0.22 μm membranes, diluted tenfold with 10% HNO₃, and analyzed by ICP-OES (iCAP PRO XP Duo, Thermo Scientific). Metal removal was calculated relative to the time-matched abiotic control as removal (%) = [(C_control, t − C_treatment, t)/C_control, t] × 100, where C_control, t is the dissolved metal concentration in the uninoculated control and C_treatment, t is the concentration in the inoculated treatment at the same sampling time. Negative calculated values were reported as 0% because they did not indicate net removal.

### Gamma irradiation and selection of tolerant derivatives

Actively growing cultures were exposed to 0, 0.5, 1, 2, 3, 4, 8, and 16 kGy gamma irradiation using a Gamma Cell CM-20 source at the Egyptian Atomic Energy Authority, Abu Zaabal, Egypt. The dose rate was 233 Gy/h verified with alanine dosimetry according to ISO/ASTM 51,261. Surviving colonies were recovered on agar plates and screened for continued growth. Because the bacterial isolate did not survive even the first non-zero dose, only the yeast derivative was taken forward for post-irradiation metal-removal experiments.

### Carotenoid extraction and HPLC analysis

Yeast pellets were subjected to thermal-acid disruption by resuspension in 2 mol/L HCl (approximately 10 g/L biomass) followed by stirring at 65 °C for 35 min. After rapid cooling and washing, pigments were extracted with absolute ethanol, then equal volume of water and n-hexane until the biomass became colorless. The combined hexane phases were dried under nitrogen and analyzed by HPLC with diode-array detection at 450 nm using a C18 column and an acetonitrile: methanol: tetrahydrofuran:1% ammonium acetate mobile phase (68:22:7:3, v/v/v/v) at 1.0 mL/min [47].

### Plasmid profiling

Plasmid DNA was extracted from the selected bacterial and yeast isolates using a QIAprep Spin Miniprep Kit (Qiagen, Germany). DNA was separated on 0.8% agarose in 1× TBE at 5 V/cm and visualized against a 50 kb ladder. The resulting bands were interpreted qualitatively as evidence of extrachromosomal DNA.

### Application to real textile saline effluents

Textile wastewater was obtained from a fabric-dyeing factory. Its initial metal concentrations, pH, and TDS are reported in Table 10. The wastewater was sterilized, supplemented with peptone (10 g/L) and yeast extract (5 g/L), and adjusted to 3.5% (w/v) total dissolved salts with NaCl. Aliquots were inoculated with 2 mL of each test culture (approximately 1.5 × 10^8 cells/mL). An uninoculated wastewater control underwent the same supplementation, sterilization, incubation, and analytical processing. Cultures and controls were incubated at 29 °C and 130 rpm for 72 h, then centrifuged, filtered, acidified, and analyzed by ICP-OES as described for the synthetic medium. Removal was calculated against the time-matched uninoculated wastewater control using the equation in Sect.  2.5.  

### Neutron activation analysis (NAA)

Harvested biomass was analyzed by neutron activation analysis (NAA) using the established irradiation and gamma-spectrometric protocol at the Egypt Second Research Reactor (ETRR-2). Samples were irradiated with neutrons, and induced gamma activity was measured using a high-purity germanium detector. Gamma spectra were used to estimate element concentrations in the recovered biomass. Because the assay was performed on harvested biomass and did not distinguish surface-bound from intracellular metal, these data are described as biomass-associated concentrations rather than intracellular uptake.

### Statistical analysis

Each reported value was obtained from triplicate analytical determinations of two independent experiments. Results expressed as mean ± standard deviation (SD). Statistical significance was assessed using one-way analysis of variance (ANOVA) followed by LSD test performed in SPSS software (version 22.0; IBM Corp., NY, USA). Differences were considered statistically significant at *P* < 0.05.

## Results and discussion

### Molecular identification of the selected isolates

**Fig. 1:**
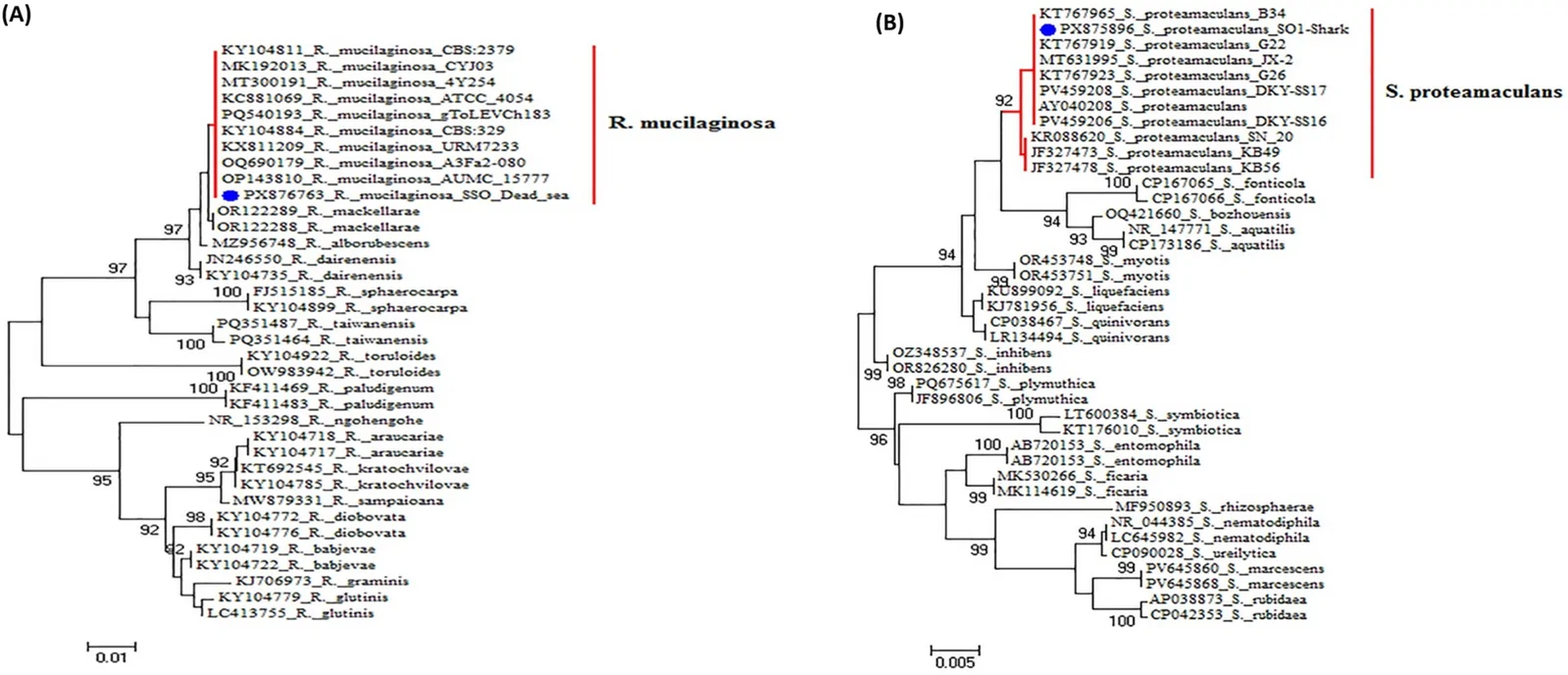
Neighbor-joining phylogenetic trees showing the placement of the selected yeast isolate with Rhodotorula mucilaginosa (**A**) and the selected bacterial isolate with Serratia proteamaculans (**B**)

Sequence-based identification placed the two most promising isolates within well-known stress-tolerant microbial groups. The yeast isolates clustered with *Rhodotorula mucilaginosa* and was assigned accession PX876763, while the bacterial isolate clustered with *Serratia proteamaculans* and was assigned accession PX875896 (Figs. 1A and B). This taxonomic outcome is reasonable for the sampled habitats. *Rhodotorula* spp. is frequently recovered from saline, metal-exposed, or otherwise stressed substrates and are valued for their carotenoid-rich phenotype and adaptive envelope chemistry [6, 31, 32, 34]. *Serratia proteamaculans* is not an obligate halophile, but members of the genus are physiologically flexible and can persist in nutrient-rich marine-associated niches, including animal surfaces. The present data therefore point to a pair of halotolerant rather than truly extreme halophilic isolates, which may be advantageous for engineered wastewater systems because such strains often remain active over a broader operational window than strict salt specialists [1, 3, 12].

### NaCl tolerance confirms moderate halotolerance

Both isolates grew at 3.0–5.0% NaCl, but neither tolerated 10% NaCl or higher under the conditions tested (Table 1). *Serratia proteamaculans* showed visible growth after 24 h at 3.0, 3.5, and 5.0% NaCl, whereas *Rhodotorulamucilaginosa* required 48 h before colonies became apparent at the same salinity range. This delayed onset in the yeast likely reflects the energetic cost of osmotic adjustment, including glycerol accumulation, membrane remodeling, and antioxidant balancing. In practical terms, the two isolates behaved as moderate halotolerants capable of functioning in mildly saline wastewater, not as extreme halophiles suited to hypersaline brines. That distinction matters because many industrial effluents fall in the low- to medium-salinity range, where moderately halotolerant strains can outperform strict specialists owing to their wider physiological plasticity [1–3, 6, 49].

**Table 1:** Growth response of the selected isolates at different NaCl concentrations

Isolate	NaCl (%)	24 h	48 h	72 h	96 h
*Serratia proteamaculans* PX875896	3.0	+	+	+	+
	3.5	+	+	+	+
	5.0	+	+	+	+
	10.0	-	-	-	-
	15.0	-	-	-	-
	20.0	-	-	-	-
	25.0	-	-	-	-
*Rhodotorula mucilaginosa* PX876763	3.0	-	+	+	+
	3.5	-	+	+	+
	5.0	-	+	+	+
	10.0	-	-	-	-
	15.0	-	-	-	-
	20.0	-	-	-	-
	25.0	-	-	-	-

*+*,* visible growth; -*,* no visible growth.*

### Metal susceptibility patterns

The inhibition assay showed different susceptibility patterns for the two isolates (Table 2). *R. mucilaginosa* showed no measurable inhibition halo up to 10 mM Co, Cu, Mo, or Cd, but showed Cr inhibition zones from 24.50 ± 0.71 mm at 10 mM to 15.00 ± 0.00 mm at 1.25 mM. *S. proteamaculans* showed no Cd inhibition halo within the tested range, inhibition by Cu and Mo only at 10 mM, and inhibition by Co and Cr over wider concentration ranges. These observations define the working concentrations used in the liquid assays but do not identify the underlying resistance mechanism.

**Table 2:** Inhibition-zone diameters (mm, mean ± SD) of the selected isolates in the presence of different metal concentrations

Concentration (mM)	S. proteamaculans					*R*. mucilaginosa				
	Co	Cu	Mo	Cr	Cd	Co	Cu	Mo	Cr	Cd
10	15.00 ± 0.00	10.50 ± 0.71	15.00 ± 0.00	19.00 ± 1.41	-	-	-	-	24.50 ± 0.71	-
5	15.50 ± 0.71	-	-	15.00 ± 1.41	-	-	-	-	24.50 ± 0.71	-
2.5	18.00 ± 1.41	-	-	17.00 ± 1.41	-	-	-	-	19.50 ± 0.71	-
1.25	15.00 ± 0.00	-	-	-	-	-	-	-	15.00 ± 0.00	-
0.625	-	-	-	-	-	-	-	-	-	-
0.313	-	-	-	-	-	-	-	-	-	-
0.16	-	-	-	-	-	-	-	-	-	-
0.078	-	-	-	-	-	-	-	-	-	-
LSD	2.45	-	-	3.78	-	-	-	-	3.44	-

- indicates that no inhibition halo was observed. Working concentrations for liquid-removal assays were selected below the lowest concentration that yielded a visible inhibition zone

### Metal removal in synthetic saline medium

When the isolates were transferred from agar screening to liquid saline medium, *S. proteamaculans* produced higher descriptive removal values than the wild yeast after 72 h: Cd, 50.71%; Mo, 42.56%; Cu, 34.13%; and Co, 25.26% (Table 3). No net Cr removal was detected. The corresponding wild-*R. mucilaginosa* values after 72 h ranged from 3.60% for Cd to 7.63% for Co. These measurements establish a performance difference under the tested conditions but do not distinguish adsorption, precipitation, extracellular binding, redox transformation, or intracellular sequestration.

**Table 3:** Removal of metals by the wild isolates in synthetic saline medium

Metal	Concentration (mM)	S. proteamaculans removal (%) at 48 h	S. proteamaculans removal (%) at 72 h	*R*. mucilaginosa removal (%) at 48 h	*R*. mucilaginosa removal (%) at 72 h	LSD
Cobalt	0.625	3.57 ± 0.05	25.26 ± 1.23	0.00	7.63 ± 0.43	1.43
Copper	5.00	0.00	34.13 ± 2.11	0.00	5.62 ± 0.22	7.98
Molybdenum	5.00	14.06 ± 0.21	42.56 ± 3.22	0.00	6.24 ± 0.19	5.88
Chromium	0.625	0.00	0.00	0.00	0.00	0.00
Cadmium	10	19.54 ± 0.76	50.71 ± 4.32	0.00	3.60 ± 0.45	2.98

### Changes in pH and dissolved solids during cultivation

The pH and TDS data support the idea that the two isolates interacted with the test medium in different ways (Table 4). In the Serratia system, pH remained around 5.28 ± 0.10 at 48 h and 5.29 ± 0.13 at 72 h, close to the acidic metal-containing control, while TDS decreased from 33120.00 ± 461.60 mg/L in the metal-only control to 29721.60 ± 1611.07 mg/L after 72 h. The *Rhodotorula* system became slightly more acidic, with pH near 4.95, and TDS remained high. These shifts accompanied the removal measurements but cannot establish whether metal loss resulted from sorption, ion exchange, precipitation, or another process. Similar medium-dependent shifts have been reported in biosorption systems where pH strongly influences metal-binding site protonation and competitive ion exchange [19, 23, 50].

**Table 4:** Changes in pH and total dissolved solids (TDS) during metal-removal experiments in synthetic saline medium

Parameter	Control: metals only	Control: isolate only	S. proteamaculans 48 h	S. proteamaculans 72 h	*R*. mucilaginosa 48 h	*R*. mucilaginosa 72 h	LSD
pH	5.21 ± 0.01	8.74 ± 0.00	5.28 ± 0.10	5.29 ± 0.13	4.95 ± 0.04	4.95 ± 0.04	1.03
TDS (mg/L)	33120.0 ± 461.6	23500.0 ± 141.4	29590.4 ± 3235.7	29721.6 ± 1611.1	33267.2 ± 2606.7	32681.6 ± 402.8	3541.22

### Gamma tolerance was confined to the carotenoid-producing yeast

Gamma irradiation sharply separated the two isolates. *Serratia proteamaculans* was not recoverable after the first non-zero dose, whereas *Rhodotorula mucilaginosa* survived up to 4 kGy, declining from 1.5 × 108 cells/mL in the unirradiated control to 5.7 × 10^2 cells/mL at 4 kGy (Table 5). No survivors were detected at 8 or 16 kGy. This survival profile demonstrates greater radioresistance of the yeast under the tested conditions. Radio-resistance in microorganisms is usually discussed in terms of DNA repair, low-protein oxidation, metal homeostasis, and small-molecule antioxidants, but pigments are also part of that picture because they quench reactive oxygen species and stabilize lipid-rich structures [41–45]. The present result also agrees with earlier yeast mutagenesis work showing that low-dose gamma exposure can preferentially yield carotenoid-rich survivors with improved oxidative fitness. These precedents make carotenoids a plausible contributor, but the present survival assay did not test carotenoid function directly.

**Table 5:** Survival of the selected isolates after gamma irradiation

Dose (kGy)	S. proteamaculans (CFU/mL)	*R*. mucilaginosa (CFU/mL)
0	1.5 × 10^8^± 0.02	1.5 × 10^8^± 0.03
0.5	0	2.9 × 10^7^± 0.01
1	0	5.39 × 10^4^± 0.12
2	0	3.3 × 10^4^± 0.11
3	0	1.74 × 10^3^± 0.05
4	0	5.7 × 10^2^± 0.02
8	0	0
16	0	0

### Gamma-selected Rhodotorula derivatives showed improved removal of several metals

Post-irradiation screening showed dose- and metal-dependent differences in dissolved-metal removal by the gamma-selected *R. mucilaginosa* derivatives (Tables 6 and 7). The highest descriptive values were Co removal by the 4 kGy derivative at 48 h (22.61%), Cu removal by the 4 kGy derivative at 72 h (12.34%), Mo removal by the 3 kGy derivative at 72 h (14.73%), and Cd removal by the 3 kGy derivative at 72 h (10.61%). NAA detected Cd, Co, Cr, Cu, and Mo in biomass recovered after metal exposure (Table 8). These are biomass-associated concentrations and cannot be interpreted as proof of intracellular uptake because surface-bound metal was not distinguished from internalized metal. Sodium concentrations decreased in exposed biomass relative to the metal-free controls, but the basis of this association was not investigated.

**Table 6:** Calculated removal by wild and gamma-irradiated *Rhodotorula mucilaginosa* derivatives in synthetic saline medium

Metal	Control (mg/L)	Wild 72 h Removal (%)	3 kGy 48 h Removal (%)	3 kGy 72 h Removal (%)	4 kGy 48 h Removal (%)	4 kGy 72 h Removal (%)	LSD
Cadmium	422.19 ± 10.55	3.69 ± 0.21	3.48 ± 0.21	10.61 ± 1.01	8.07 ± 0.51	9.10 ± 0.32	1.32
Cobalt	643.44 ± 45.22	7.64 ± 0.33	16.51 ± 0.76	16.39 ± 0.54	22.61 ± 0.87	19.05 ± 0.98	3.11
Chromium	41.94 ± 2.98	0.0	0.0	0.0	0.0	0.0	-
Copper	467.64 ± 11.45	6.23 ± 0.22	6.91 ± 0.10	8.85 ± 0.21	10.63 ± 0.54	12.34 ± 0.87	1.09
Molybdenum	1465.41 ± 67.55	6.27 ± 0.21	4.93 ± 0.21	14.73 ± 0.32	12.87 ± 0.31	9.11 ± 0.65	1.01

These differences are relative to a weakly active parent yeast. Gamma selection was associated with altered metal-removal performance, but the responsible cellular mechanism remains undetermined. Mutation-assisted phenotype improvement is well established in carotenoid yeasts, and in Rhodotorula the resulting gain is often broader than pigment yield alone because oxidative-stress management, membrane composition, and metabolite trafficking are physiologically linked [37, 48]. Chromium behaved differently: residual Cr concentrations in treated flasks exceeded the measured control, so no net chromium removal can be claimed under these conditions. That result may reflect oxidation-state changes, desorption, or analytical variation, and it warns against treating all metals as mechanistically equivalent in mixed systems [21, 22, 26, 27].

**Table 7:** pH and TDS changes during the gamma-irradiated yeast experiment

Parameter	Control	Wild 72 h	3 kGy 48 h	3 kGy 72 h	4 kGy 48 h	4 kGy 72 h	LSD
pH	5.11 ± 0.01	4.95 ± 0.04	4.80 ± 0.00	4.71 ± 0.01	4.92 ± 0.00	4.74 ± 0.12	0.982
TDS (mg/L)	31523.2 ± 1117.8	32681.6 ± 402.8	33875.0 ± 117.4	31637.0 ± 2694.1	33472.0 ± 90.5	33453.0 ± 434.2	5432.04

### Carotenoid profiles of wild and gamma-selected yeast

**Fig. 2:**
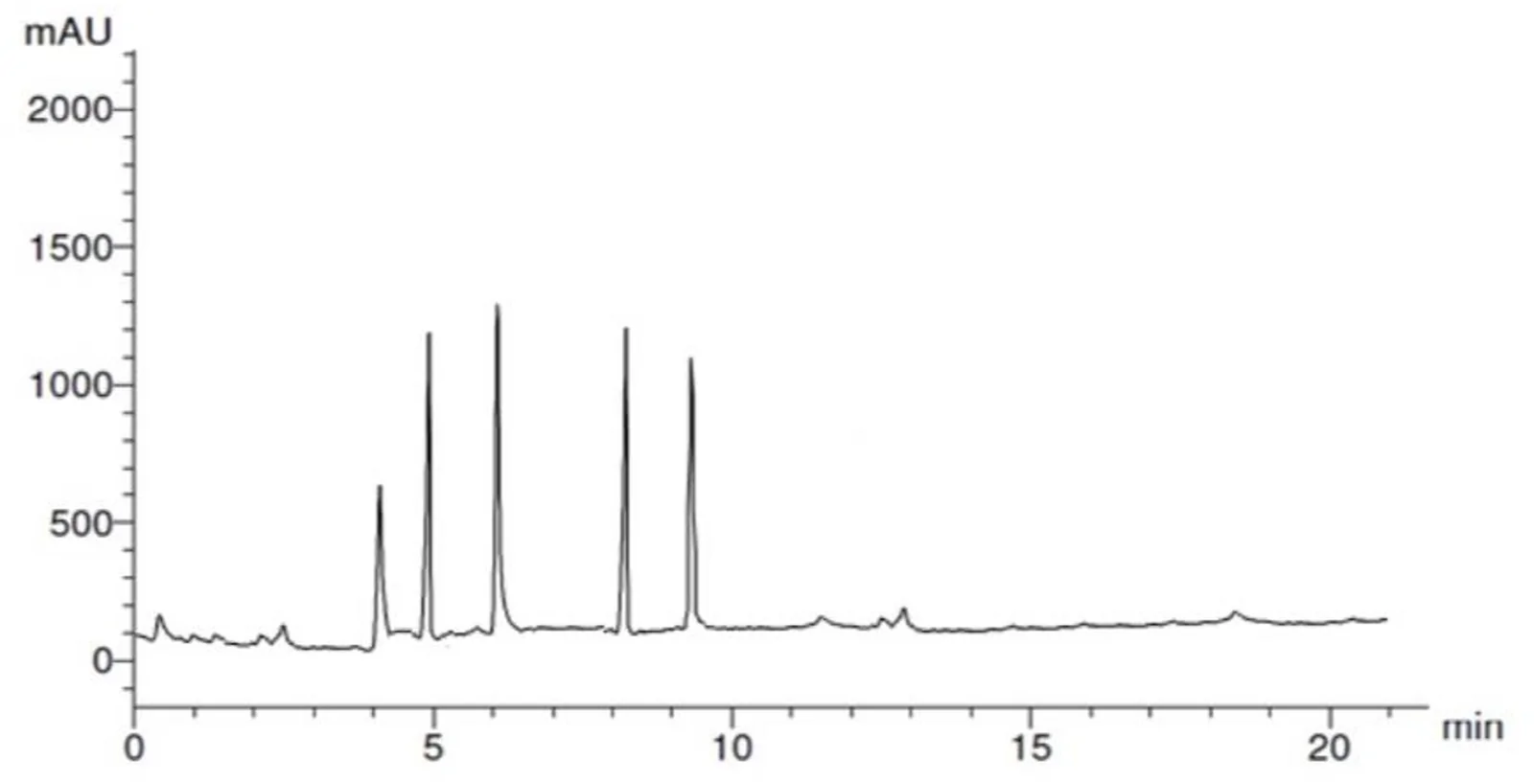
Representative HPLC chromatograms of carotenoids extracted from the wild strain and the two gamma-selected yeast derivatives

Carotenoid profiles differed among the wild strain and gamma-selected derivatives (Table 9; Fig. 2). Total quantified carotenoids were 44.40 µg/mL in the wild strain, 41.98 µg/mL in the 3 kGy derivative, and 67.30 µg/mL in the 4 kGy derivative. Torularhodin was not detected in the 3 kGy derivative but reached 19.00 µg/mL in the 4 kGy derivative. β-Carotene was 14.62 µg/mL in the wild strain and 16.98 µg/mL in the 4 kGy derivative. These measurements demonstrate an association between selection at 4 kGy, survival, and a carotenoid-rich profile. They do not show that carotenoid enrichment caused radioresistance, because carotenoid depletion, pathway inhibition, gene-expression analysis, or genetic complementation was not performed.

### Plasmid profiles

**Fig. 3:**
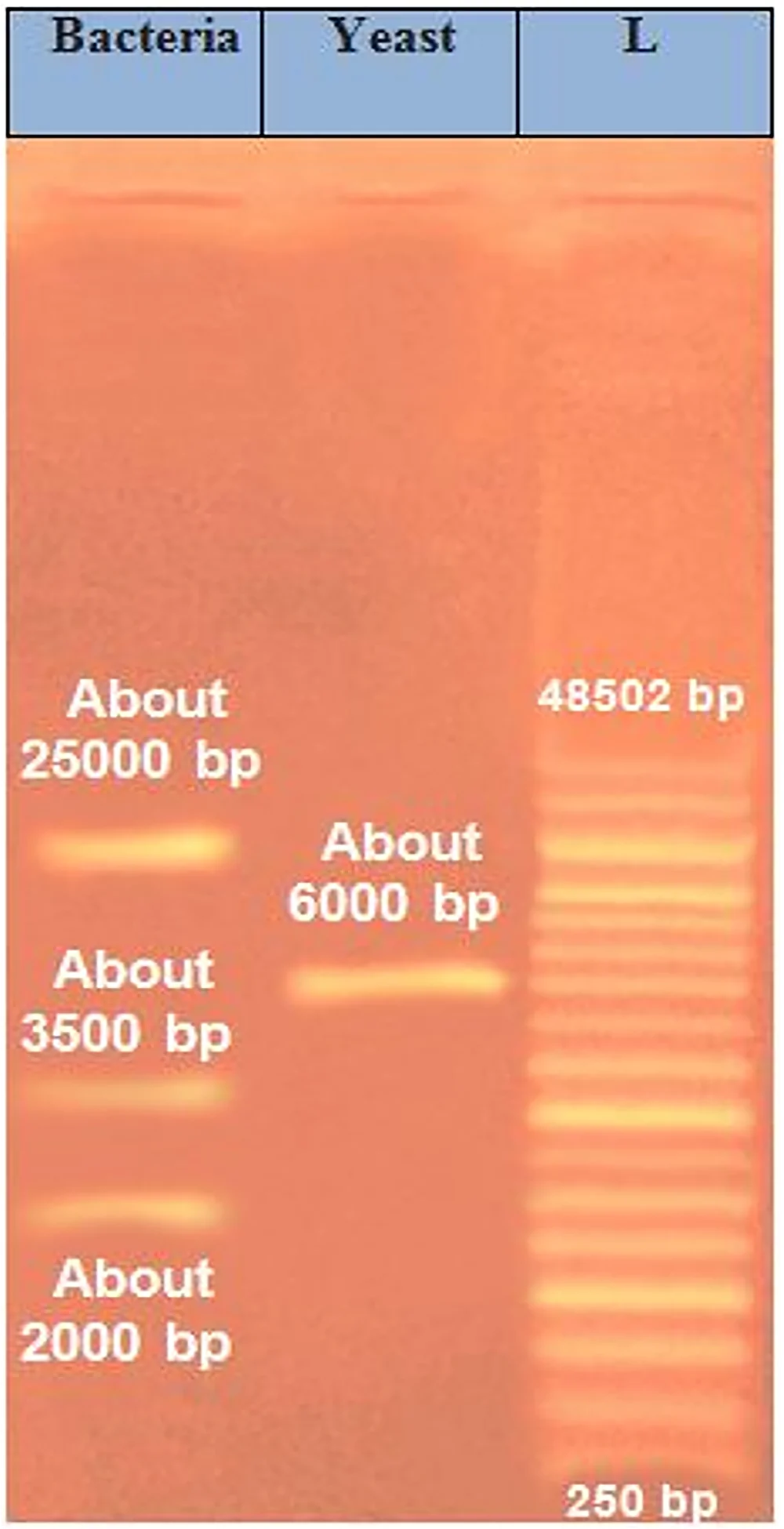
Plasmid profile of the selected bacterial and yeast isolates showing discrete extrachromosomal DNA bands relative to the ladder (L)

The plasmid gel showed discrete extrachromosomal bands in both isolates, with multiple bands in the bacterial sample and a single prominent band in the yeast sample (Fig. 3). Although no sequencing was performed, the presence of plasmids is worth noting because accessory DNA is often associated with stress tolerance, metal resistance determinants, transport systems, and horizontal acquisition of adaptive traits in contaminated environments.

**Table 8:** Biomass-associated element concentrations measured by NAA

Wild and gamma-irradiated Rhodotorulamucilaginosa derivatives						
Metal	Control (g/ Kg)	Wild 72 h (g/Kg)	3 kGy 72 h (g/ Kg)	4 kGy 72 h (g/ Kg)	LSD	
Cadmium	NA	12.2 ± 0.48	7.60 ± 1.24	13.4 ± 0.52	0.87	
Cobalt	NA	5.34 ± 0.16	4.54 ± 0.16	4.61 ± 0.19	0.67	
Chromium	NA	7.20 ± 0.27	12.7 ± 0.29	14.4 ± 0.27	1.02	
Copper	NA	10.8 ± 7.97	24.2 ± 17.74	25.7 ± 9.75	1.98	
Molybdenum	NA	48.3 ± 0.3	66.1 ± 0.56	74.7 ± 0.24	10.99	
Sodium	61.8 ± 0.015	57.4 ± 0.25	26.2 ± 0.41	16.8 ± 0.52	4.95	
**Wild and gamma-irradiated** ***S. proteamaculans***						
**Metal**	**Control** **(g/Kg)**	**Wild 48 h** **(g/Kg)**	**Wild 72 h (g/Kg)**	**LSD**		
Cadmium	NA	25.7 ± 0.78	31.3 ± 0.25	3.76	—	—
Cobalt	NA	0.942 ± 1.46	1.55 ± 0.47	0.06	—	—
Chromium	NA	13.4 ± 1.2	12.9 ± 0.59	0.98	—	—
Copper	NA	32.0 ± 6.9	26.3 ± 6.93	0.78	—	—
Molybdenum	NA	57.6 ± 1.12	55.7 ± 0.46	4.32	—	—
Sodium	51.2 ± 0.2	8.38 ± 1.48	10.7 ± 0.56	2.76	—	—

### Real textile wastewater favored the irradiated yeast derivatives

The textile wastewater contained B, Cd, Co, Cr, Cu, Fe, Mn, Mo, Sr, V, and Zn; its initial composition, pH, and TDS are reported in Table 10. Table 11 reports removal percentages and calculated residual concentrations after 72 h relative to the time-matched uninoculated control. The 4 kGy yeast derivative produced the highest removal values for Mo (93.05%; residual 0.150 mg/L), Cr (77.88%; 0.024 mg/L),Co (60.80%; 0.034mg/L), Cd (51.42%; 0.077 mg/L), Cu (22.44%; 0.194), B (14.38%; 0.313 mg/L), Sr (3.98%; 0.289 mg/L), and Zn (16.59%; 0.284 mg/L). The 3 kGy derivative gave the highest Cr ( 74.65; 0.027 mg/L) and Co removal (72.73%; 0.024 mg/L). *S. proteamaculans* gave the highest removal of Mo (58.43 %; 0.895 mg/L) , Cr ( 57.14; 0.046 mg/L) , Cu (45.67%; 0.138 mg/L), Fe (52.17%; 0.132 mg/L), and V (74.68%; 0.010 mg/L). Table 12 presents the pH and TDS measured after textile-wastewater treatment with S. proteamaculans, wild R. mucilaginosa, and the 3 and 4 kGy yeast derivatives. Treatment increased the pH from 6.03 in the control to 8.62–8.25 across the biological treatments, while TDS ranged from 29,997.0 to 31,002.0 mg/L compared with 30,547.0 mg/L in the control.

**Table 9:** Carotenoid composition of wild and irradiated *Rhodotorula mucilaginosa* derivatives

Carotenoid	Retention time (min)	Wild (µg/mL)	3 kGy (µg/mL)	4 kGy (µg/mL)
Torularhodin	2.0	5.02	ND	19.0
β-Carotene	5.0	14.62	9.52	16.98
Torulene	6.0	6.56	13.74	12.85
Lutein	7.9	7.01	10.09	10.14
γ-Carotene	9.0	11.19	8.63	8.33
Total carotenoids	—	44.4	41.98	67.3

*ND*,* not detected. Total carotenoids were calculated from the quantified compounds listed in the table.*

The present work shows that marine-associated samples can yield functionally distinct microorganisms with real promise for metal cleanup. *Serratia proteamaculans* PX875896 and *Rhodotorula mucilaginosa* PX876763 were both moderately halotolerant, but they behaved very differently under metal and radiation stress. The bacterial isolate was the better remover in synthetic saline medium, especially for cadmium, molybdenum, and copper, whereas the yeast displayed broader metal tolerance and substantially greater resistance to gamma irradiation. Selection of irradiated *Rhodotorula* derivatives, particularly the 4 kGy variant, improved multimetal removal in real textile wastewater and coincided with a shift toward a more carotenoid-rich phenotype. These findings support four practical conclusions. First, moderate halotolerance can still be highly useful for treatment of saline industrial effluents; extreme halophily is not always required. Second, strain tolerance alone is not a reliable predictor of short-term removal efficiency. Third, gamma selection can generate yeast derivatives with altered metal-removal phenotypes. Fourth, saline wastewater treatment may benefit from complementary use of bacterial and yeast isolates rather than reliance on a single organism. Future work should validate these strains in continuous systems, resolve chromium speciation during treatment, sequence the irradiated derivatives to identify the genetic basis of the improved phenotype, and evaluate whether a bacterial-yeast consortium can deliver both rapid primary removal and stable downstream polishing.

**Table 10:** Chemical characteristics of the textile wastewater used for the application experiment

Parameter	Textile wastewater (mg/L)
Boron	0.365 ± 0.014
Cadmium	0.159 ± 0.073
Cobalt	0.088 ± 0.007
Chromium	0.108 ± 0.021
Copper	0.254 ± 0.095
Iron	0.277 ± 0.040
Manganese	0.013 ± 0.003
Molybdenum	2.152 ± 0.328
Strontium	0.301 ± 0.005
Vanadium	0.040 ± 0.006
Zinc	0.341 ± 0.074
pH	6.03 ± 0.65
TDS (mg/L)	30547.0 ± 1357.6

Taken together, the study does not point to a single universally superior organism. Instead, it reveals two complementary tools. Wild *Serratia proteamaculans* was the better fast-acting remover in defined saline medium, particularly for Cd, Mo, and Cu. Wild *Rhodotorula mucilaginosa* was less efficient at first, yet it alone tolerated gamma irradiation and, after selection, became the stronger candidate for real mixed-metal wastewater. That division is scientifically sensible. In engineered remediation, robustness under process stress can matter more than peak performance in a simplified flask test. For saline multimetal treatment, the most useful future strategy may therefore be a staged or consortium-based system combining rapid bacterial binding with stress-tolerant yeast polishing. Such a framework is compatible with recent thinking on extremophile-assisted remediation, adaptive strain improvement, and mixed consortia designed to distribute functions across adsorption, detoxification, and polishing steps [18, 50, 51].

**Table 11:** Removal of metals from textile wastewater by the bacterial isolate, wild yeast, and irradiated yeast derivatives

Metal	Control (mg/L)	Bacterial treatment: removal (%); residual (mg/L)	Wild yeast: removal (%); residual (mg/L)	3 kGy yeast: removal (%); residual (mg/L)	4 kGy yeast: removal (%); residual (mg/L)	LSD
Cadmium	0.159 ± 0.073	44.16%; 0.089 mg/L	0.00%; 0.159 mg/L	0.00%; 0.159 mg/L	51.42%; 0.077 mg/L	1.87
Cobalt	0.088 ± 0.007	32.39%; 0.059 mg/L	0.00%; 0.088 mg/L	72.73%; 0.024 mg/L	60.80%; 0.034 mg/L	2.54
Chromium	0.108 ± 0.021	57.14%; 0.046 mg/L	38.25%; 0.067 mg/L	74.65%; 0.027 mg/L	77.88%; 0.024 mg/L	4.98
Copper	0.254 ± 0.095	45.67%; 0.138 mg/L	0.00%; 0.254 mg/L	20.67%; 0.201 mg/L	22.44%; 0.197 mg/L	3.98
Molybdenum	2.152 ± 0.328	58.43%; 0.895 mg/L	18.75%; 1.749 mg/L	45.47%; 1.173 mg/L	93.05%; 0.150 mg/L	2.74
Boron	0.365 ± 0.014	0.00%; 0.365 mg/L	8.77%; 0.333 mg/L	0.00%; 0.365 mg/L	14.38%; 0.313 mg/L	1.98
Iron	0.277 ± 0.040	52.17%; 0.132 mg/L	22.38%; 0.215 mg/L	27.80%; 0.200 mg/L	40.43%; 0.165 mg/L	4.87
Manganese	0.012 ± 0.003	16.67%; 0.010 mg/L	16.67%; 0.010 mg/L	16.67%; 0.010 mg/L	16.67%; 0.010 mg/L	2.18
Strontium	0.301 ± 0.005	1.16%; 0.298 mg/L	0.33%; 0.300 mg/L	3.32%; 0.291 mg/L	3.98%; 0.289 mg/L	1.00
Vanadium	0.040 ± 0.006	74.68%; 0.010 mg/L	10.13%; 0.036 mg/L	0.00%; 0.040 mg/L	2.53%; 0.039 mg/L	1.87
Zinc	0.341 ± 0.074	6.46%; 0.319 mg/L	7.78%; 0.314 mg/L	11.31%; 0.302 mg/L	16.59%; 0.284 mg/L	2.09

**Table 12:** pH and TDS after textile-wastewater treatment

Parameter	Control	Bacteria	Wild yeast	3 kGy yeast	4 kGy yeast	LSD
pH	6.03 ± 0.65	8.62 ± 0.00	8.39 ± 0.11	8.21 ± 0.00	8.25 ± 0.01	0.981
TDS (mg/L)	30547.0 ± 1357.6	31002.0 ± 0.0	30390.5 ± 330.2	29997.0 ± 1014.0	30045.0 ± 176.8	2343.21

## Conclusions

*Serratia proteamaculans* PX875896 and *Rhodotorula mucilaginosa* PX876763 were moderately halotolerant, growing at 3–5% NaCl but not at 10% or higher. *S. proteamaculans* yielded greater dissolved-metal removal than the wild yeast in synthetic saline medium, whereas only *R. mucilaginosa* survived gamma exposure up to 4 kGy. Gamma-selected yeast derivatives showed altered, metal-dependent removal and carotenoid profiles, and the 4 kGy derivative produced high descriptive removal values for several metals in textile wastewater. The carotenoid and survival data are correlative, and neither the dissolved-metal measurements nor NAA establish a specific removal mechanism. Complete mass-balance analysis and mechanistic validation are required before process-level conclusions or scale-up claims can be made.

## Supplementary Information

Below is the link to the electronic supplementary material.

fig 2 wild

fig2 3k

fig2- 4k

## Data Availability

The nucleotide sequences generated in this study are available in GenBank under accession numbers PX875896 and PX876763. https://www.ncbi.nlm.nih.gov/nuccore/PX876763https://www.ncbi.nlm.nih.gov/nuccore/PX875896.

## Abbreviations

CFU: colony-forming units
HPLC: high-performance liquid chromatography
ICP-OES: inductively coupled plasma optical emission spectrometry
NAA: neutron activation analysis
TDS: total dissolved solids
